# The genome of the cotton bacterial blight pathogen *Xanthomonas citri* pv. *malvacearum* strain MSCT1

**DOI:** 10.1186/s40793-017-0253-3

**Published:** 2017-07-24

**Authors:** Kurt C. Showmaker, Mark A. Arick, Chuan-Yu Hsu, Brigitte E. Martin, Xiaoqiang Wang, Jiayuan Jia, Martin J. Wubben, Robert L. Nichols, Tom W. Allen, Daniel G. Peterson, Shi-En Lu

**Affiliations:** 1 Institute for Genomics, Biocomputing and Biotechnology, Mississippi State University, Mississippi State, MS 39762 USA; 2 Department of Biochemistry, Molecular Biology, Entomology and Plant Pathology, Mississippi State University, Mississippi State, MS 39762 USA; 3 Department of Basic Sciences, College of Veterinary Medicine, Mississippi State University, Mississippi State, MS 39762 USA; 4 USDA-ARS, Crop Science Research Lab, Genetics and Sustainable Agriculture Research Unit, Mississippi State, MS 39762 USA; 5 Cotton Incorporated, Cary, NC 27513 USA; 6 Mississippi State University, Delta Research and Extension Center, 82 Stoneville Rd, Stoneville, MS 38776 USA; 7 Department of Plant & Soil Sciences, Mississippi State University, Mississippi State, MS 39762 USA

**Keywords:** *Xanthomonas citri* pv. *malvacearum*, Bacterial blight, TAL effectors, Cotton, Long read DNA sequencing

## Abstract

*Xanthomonas citri* pv. *malvacearum* is a major pathogen of cotton, *Gossypium hirsutum* L.. In this study we report the complete genome of the *X. citri* pv. *malvacearum* strain MSCT1 assembled from long read DNA sequencing technology. The MSCT1 genome is the first *X. citri* pv. *malvacearum* genome with complete coding regions for *X. citri* pv. *malvacearum* transcriptional activator-like effectors. In addition functional and structural annotations are presented in this study that will provide a foundation for future pathogenesis studies with MSCT1.

## Introduction

*Xanthomonas citri* pv. *malvacearum* is the causal agent of bacterial blight of cotton (*Gossypium hirsutum* L.). *Xanthomonas citri* pv. *malvacearum* infects plant tissues and organs of cotton during all stages of development beginning with the seedling stage [1]. Typical disease symptoms caused by *X. citri* pv. *malvacearum* include cotyledon/seedling blight, angular leaf spot, systemic vein blight, black arm (of petioles and main stems), boll shedding, and internal boll rot [1]. Histology studies reported that the host cotton plant cells begin to degenerate 3 days post-infection [2]. Over the 3 day period the degradation of host cells begins by; first, the host tissue appearing to loosen, then the granal and stromal membranes of the chloroplasts disappear, followed by the degeneration of the chloroplast and other organelles [2, 3]. At 6 days post-infection, cellular degeneration along with the production of a hydrophilic extracellular polymeric substance by the bacterium, causes water to accumulate in the infected tissues forming lesions known as “water soaked spots”, a classical plant pathogen-associated symptom [2–4].

Resistance to *X. citri* pv. *malvacearum* has been identified in cotton, as well as additional *Gossypium* species. Currently, most lines resistant to *X. citri* pv. *malvacearum* exist in *G. hirsutum* cultivars since breeding for *X. citri* pv. *malvacearum* resistance has been ongoing since 1939 [5] and continues today as *G. hirsutum* cultivars and germplasm releases are screened for *X. citri* pv. *malvacearum* resistance [6–8]. At least 18 genes participate in resistance to *X. citri* pv. *malvacearum* [1, 9]. The ability of the *X. citri* pv. *malvacearum* strains to escape specific resistance genes resulted in a classification scheme of races. To date, 22 races have been reported and assigned numerical names (i.e. 1 to 22) [9]. Most races are geographically distinct. Of note, bacterial blight in the U.S. is predominantly caused by race 18. Genetic resistance within cotton cultivars is generally attributed to a certain race or multiple races of *X. citri* pv. *malvacearum*. The ability of *G. hirsutum* to mount a defense response to *X. citri* pv. *malvacearum* is, at least in some cases, dependent upon the transcription activator-like effector avrBs3/pthA gene family in *X. citri* pv. *malvacearum* indicating the presence of a gene-for-gene relationship in *X. citri* pv. *malvacearum*-*G. hirsutum* interactions [9, 10]. With the ever increasing understanding of the importance of TAL effectors in pathogenesis [11–13], the objective of this study was to generate the first genome sequence for a *X. citri* pv. *malvacearum* strain that contains the TAL effector complement to serve as a foundation for a better understanding of the *X. citri* pv. *malvacearum*-*G. hirsutum* interaction.

To date, four draft genomes of *Xanthomonas citri* pv. *malvacearum* have been published. However, all sequenced *X. citri* pv. *malvacearum* isolates were obtained from outside the United States [14, 15]. The diversity of the four previously reported draft genomes includes two race 18 isolates, one race 20 isolate, and a highly virulent strain. The project described here was undertaken to provide the first *X. citri* pv. *malvacearum* genome sequence from the Mid-South region of the United States, a major production area of upland cotton. The isolate, MSCT1, was isolated during the 2011 outbreak of *X. citri* pv. *malvacearum* in the Mississippi Delta (i.e. Mississippi river’s alluvial plain). This outbreak resulted in the greatest estimated *X. citri* pv. *malvacearum*-based losses (52,000 bales) in Arkansas and Mississippi as reported by the National Cotton Council Disease Database [16]. This study was undertaken to generate a genome sequence for the *X. citri* pv. *malvacearum* strain MSCT1 to identify protein candidates that may be involved in the pathogenesis of bacteria bight of cotton. The genome sequence will also serve as a template for which further studies of genetic diversity of *X. citri* pv. *malvacearum* in the United States can be conducted.

## Organism information

### Classification and features

*Xanthomonas citri* pv. *malvacearum* has gone through a series of name changes over time as additional information has been learned about its biology and genetics. In chronological order, *X. citri* pv. *malvacearum* has previously been classified as *Pseudomonas* *malvacearum*, *Bacterium malvacearum*, and *Xanthomonas* *malvacearum* [9]. In 2009, Ah-You et al. assigned the *X. citri* pv. *malvacearum* moniker [9, 17]. *Xanthomonas citri* pv. *malvacearum* is a motile, Gram-negative, rod-shaped bacterium that produces yellow, copiously mucoid, wet, shining growth on 2% *w*/*v* peptone-sugar agar [1]. *Xanthomonas citri* pv. *malvacearum*, like other *Xanthomonas* species (xanthomonads), produces the heteropolysaccharide xanthan [4]. Additional characteristics of *X. citri* pv. *malvacearum* are provided in Table 1.

**Table 1:** Classification and general features of *Xanthomonas citri* pv. *malvacearum* strain: MSCT1 [75]

MIGS ID	Property	Term	Evidence code^a^
	Classification	Domain *Bacteria*	TAS [76]
		Phylum *Proteobacteria*	TAS [77]
		Class *Gammaproteobacteria*	TAS [78]
		Order *“Xanthomonadales”*	TAS [79]
		Family *“Xanthomonadaceae”*	TAS [79]
		Genus *Xanthomonas*	TAS [80]
		Species *Xanthomonas citri*	TAS [17]
		Pathovar *malvacearum* strain: MSCT1	
	Gram stain	*Negative*	TAS [1]
	Cell shape	*Rod*	TAS [1]
	Motility	*Motile*	TAS [1]
	Sporulation	*Not reported*	
	Temperature range	*10-38 °C*	TAS [1, 81]
	Optimum temperature	*25-30 °C*	TAS [1, 81]
	pH range; Optimum	*Optimum 6.0*	TAS [1]
	Carbon source	*Glucose, sucrose, fructose, arabinose, galactose, maltose, cellobiose, and glycerol*	TAS [1]
MIGS-6	Habitat	*Plant-associated*	TAS [1]
MIGS-6.3	Salinity	*Not reported*	
MIGS-22	Oxygen requirement	*Not reported*	
MIGS-15	Biotic relationship	*Parasitic*	TAS [1]
MIGS-14	Pathogenicity	*Pathogenic*	IDA
MIGS-4	Geographic location	*Mississippi, USA*	IDA
MIGS-5	Sample collection	*2011*	IDA
MIGS-4.1	Latitude	*Not Reported*	
MIGS-4.2	Longitude	*Not Reported*	
MIGS-4.4	Altitude	*Not Reported*	

^a^Evidence codes - *IDA* inferred from direct assay, *TAS* traceable author statement (i.e., a direct report exists in the literature), *NAS* non-traceable author statement (i.e., not directly observed for the living, isolated sample, but based on a generally accepted property for the species, or anecdotal evidence). These evidence codes are from the Gene Ontology project [82]

**Fig. 1:**
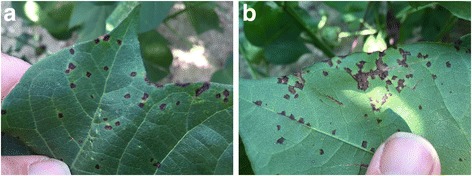
*Top* (**a**) and *bottom* (**b**) of a cotton leaf displaying the bacterial blight disease symptom caused by *Xanthomonas citri* pv. *malvacearum*

**Fig. 2:**
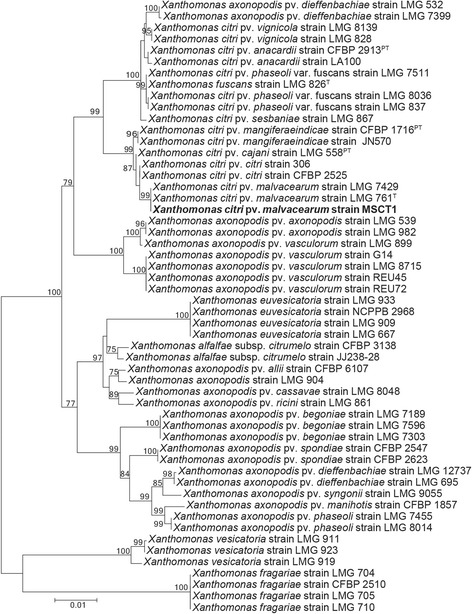
The phylogenetic tree indicating current placement of the source organism. The phylogenetic tree was constructed based on the sequences of genes coding for ATP synthase β chain (*atpD*), heat shock protein 70 (*dnaK*), and gyrase subunit β (*gyrB*) for *Xanthomonas* species. MAFFT (version 7) [85] was used to align the sequences; the evolutionary history was inferred by using the Maximum Likelihood, with 100 bootstraps, method based on the Tamura-Nei model [86] with MEGA6 [87] software. Sequence identifiers of each subunit are as reported by Ah-You et al. 2009 [17]. Type (T) and Pathovar Type (PT) strains are noted in superscript

**Fig. 3:**
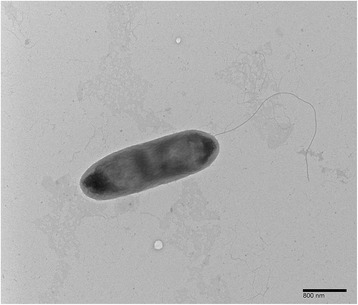
Transmission election micrograph of *Xanthomonas citri* pv. *malvacearum* strain MSCT1

For specimen isolation, cotton leaves with the typical blight symptoms (Fig. 1) were collected from a field located north of Yazoo City, Mississippi in Yazoo County, during the 2011 growing season. Strain MSCT1 was isolated using a routine method for foliar bacterial pathogens. In brief, the disease lesions were cut into small pieces (3 × 3 mm) from the junction of diseased and healthy tissues. The cut pieces were transferred into a sterile 1.5 ml microcentrifuge tube and surface-sterilized using 10% sodium hypochlorite (bleach; Clorox) for 1 min. The sterilized tissues were washed twice using sterile water, and then stabbed with a sterile lab needle in 200 μl of sterile water. A full loop of the resulting bacterial suspension was streaked on nutrient broth-yeast extract plates [18]. The streaked nutrient broth-yeast extract plates were incubated at 20 °C for 2 days under ambient laboratory temperatures and a 16:8 day: night photoperiod. Single colonies of the resulting bacterium were isolated in a sterilized loop and streaked onto fresh NBY plates for purification. The pathogenicity of MSCT1 to cause bacterial blight of cotton was confirmed by fulfilling Koch’s Postulates. Briefly, cotton seedlings (cotton cultivar PHY499WRF) were grown in the greenhouse until they reached the three-leaf growth stage. A vacuum system (20″ psi for 10 s) was used to inoculate the seedling leaves with a suspension of MSCT1 (OD 0.3 at 420 nm) suspended in sterile phosphate buffer (0.01 M; pH 7.0). After 10 days the characteristic symptoms of bacterial blight were observed on the inoculated leaf tissues. The *X. citri* pv. *malvacearum* strain MSCT1 that is described in this manuscript was deposited in the USDA Agricultural Research Service Culture Collection under deposition number NRRL B-65440. The isolate MSCT1 was confirmed to be *Xanthomonas citri* pv. *malvacearum* based on the 16S rRNA sequence analysis, as described previously [19]. Multilocus sequence typing was used to construct a phylogenic tree for *Xanthomonas* strains based upon three genes from the MLST described by Ah-You et al. 2009 [17], and included; *atpD* coding ATP synthase β chain, *dnaK* coding heat shock protein 70, and *gyrB* coding the gyrase subunit β (Fig. 2). A transmission election micrograph of MSCT1 was generated by the Mississippi State University’s Institute for Imaging & Analytical Technologies (Fig. 3).

## Genome sequencing information

### Genome project history

The MSCT1 sequencing project arose from the 2011 outbreak of bacterial blight in the cotton growing regions of the Mississippi Delta. Following MSCT1 isolation, additional testing determined that MSCT1 was capable of producing disease symptoms on several cultivars of upland cotton commonly grown in the Mid-South. Preliminary bioinformatics investigations determined *X. citri* pv. *malvacearum* assemblies, generated from short reads, lacked detectable TAL effectors in their genomes, although TAL effectors have been previously described as occurring in *X. citri* pv. *malvacearum* [20–22]. To better understand the pathology of *X. citri* pv. *malvacearum*, and more specifically of isolate MSCT1, we conducted a long read genome sequencing project to identify MSCT1’s effector complement, including the TAL effectors that do not assemble well with short read DNA sequencing technology. The resultant complete genome sequence has been deposited in the NCBI genome database under genome assembly accession GCF_001719155.1.

### Growth conditions and genomic DNA preparation

An MSCT1 colony was isolated from a LB plate (pectone 10 g/L, yeast extract 5 g/L sodium chloride 10 g/ L, agarose 15 g/L) and used to inoculate 1.5 ml of LB medium (pectone 10 g/L, yeast extract 5 g/L sodium chloride 10 g/L) in a sterile, plastic culture tube. The culture tube was placed at 25 °C with 200 rpm orbital shaking overnight. The resulting bacterial culture was pelleted by centrifugation at 5000 rpm for 10 min. The pellet was washed twice to remove LB medium; each wash consisted of resuspending the pellet in 1 ml of phosphate buffered saline (PBS; NaCL 8 g/L, KCl 0.2 g/L, Na2HPO4 1.42 g/L, KH2PO4 0.24 g/L), centrifuging the suspension at 5000 rpm for 10 min, and discarding the supernatant. Genomic DNA was isolated using a modified version of the method described in Chen and Kuo 1993 [23]. Briefly, the cell pellet was resuspended in 300 μl of extraction buffer (40 mM Tris-HCl, 1 mM EDTA, 1% *w*/*v* SDS, pH 7.8). After adding 50 μl of 10 mg/ml lysozyme (Sigma-Aldrich; St. Louis, MO, USA), the cell suspension was incubated at 37 °C for 30 min with occasional mixing until the cell suspension became clear. The bacterial nucleic acid sample was further purified using a series of phenol, phenol/chloroform, and chloroform extraction steps, then precipitated with two volumes of 100% ethanol. DNA was pelleted by centrifugation at 12,000 rpm for 10 min. After two washes with 70% ethanol (*v*/v), the nucleic acid pellet was air-dried (approximately 15 min). The pellet was then dissolved in 50 μl of 10 mM Tris buffer (pH 7.5). The bacterial nucleic acid sample was treated with 20 μl of 30 mg/ml RNase A (Sigma-Aldrich; St. Louis, MO, USA) at 37 °C for 20 min, followed by phenol/chloroform and chloroform extraction steps to remove the enzyme. The DNA was precipitated with 100% ethanol and cleaned with 70% ethanol as described above. The air-dried genomic DNA pellet was dissolved in 50 μl of 10 mM Tris buffer (pH 7.5). The resultant DNA was visualized on a 0.8% *w*/*v* agarose gel.

### Genome sequencing and assembly

Two long read technologies, PacBio (Pacific Biosciences of California, Melon Park, CA, USA) and Nanopore (Oxford Nanopore Technologies, Oxford, UK), were used to sequence MSCT1. A 20 kb PacBio library was prepared and sequenced on two P6-C4 SRMT cells at the University of Delaware Sequencing & Genotyping Center (Newark, DE, USA). Additionally, a Nanopore library was prepared and sequenced on a R9 Nanopore flowcell at the Mississippi State Institute for Genomics, Biocomputing, and Biotechnology (Mississippi State, MS, USA). The PacBio and Nanopore reads were assembled with the Canu long read assembler [24]. The resultant contigs from the assembly were aligned against themselves with blastn to identify the overlapping ends of the assembly for circularization of the DNA molecules. Following circularization, open reading frames (ORFs) were predicted with the getorf program within the ESBOSS software package [25] and the *dnaA* coding region for the protein was identified with blastn [26]. The chromosome was rearranged to place the start of the molecule 41 bp from the start of the *dnaA* coding region. The plasmid molecules were rearranged to put the resultant ends of the circularization within the middle of the molecule while allowing the new cut sight to fall outside a predicted ORF. To ensure the circulation was correct PacBio reads longer than 4000 bp were aligned to the circularized assembly with blasr [27] and manually checked with IGV [28, 29]. For additional error correction, an Illumina PCR-free DNA library with a DNA insert size of 416 bp was prepared at the Institute of Genomics, Biocomputing and Biotechnology (Mississippi State, MS, USA). The Illumina library was paired-end sequenced (2 × 300 bp) using the Illumina MiSeq. The short read pairs were trimmed with Trimmomatic [30] and subsequently used to error correct the Canu assembly with Pilon [31]. After Pilon error correction, the resultant assembly was polished with 20 kb PacBio reads using the Quiver algorithm within the PacBio SMRT Analysis software suite (version 2.3.0.140936, Pacific Biosciences of California). The Minimum Information about a Genome Sequence specification was used to report the MSCT1 genome sequencing and assembly methods (Table 2) [32].

**Table 2:** Project information

MIGS ID	Property	Term
MIGS 31	Finishing quality	Complete genome
MIGS-28	Libraries used	Paired-end (Illumina), Pacbio 20 kb, Nanopore
MIGS 29	Sequencing platforms	Illumina MiSeq, PacBio, Nanopore
MIGS 31.2	Fold coverage	2378.74X Total, 1820.26X Illumina, 516.58 PacBio, 41.90 Nanopore
MIGS 30	Assemblers	Canu v1.3, Pilon v1.17, Quiver v2.3.0
MIGS 32	Gene calling method	NCBI Prokaryotic Genome Annotation Pipeline
	Locus Tag	BGK55
	Genbank ID	GCA001719145.1
	GenBank Date of Release	06-SEP-2016
	GOLD ID	Gp0177725
	BIOPROJECT	PRJNA299817
MIGS 13	Source Material Identifier	MSCT1
	Project relevance	Agricultural

### Genome annotation

Proteins and noncoding RNAs (including rRNA, tRNA, ncRNA) were predicted with the NCBI Prokaryote Genome Annotation Pipeline [33]. Clusters of Orthologous Groups annotation of the predicted proteins against the COG position-specific scoring matrices downloaded from the NCBI Conserved Domain Database was conducted with RSP-BLAST [34–36]. InterProScan V51.0 was used to add Pfam annotations using the Pfam applet [37]. Signal peptides and transmembrane helices were predicted with SignalP [38] and TMHMM [39], respectively. Clustered regularly-interspaced short palindromic repeats sequences were identified using CRISPRFinder [40]. Plant inducible promoter sequences in the promoter region (both strands) of genes were identified with the regular expression ‘TTCGN [16] TTCG’, where N is any nucleotide, as described by Lee et al. 2005 [41–43]. EffectiveDB was used to determine if MSCT1 contains functional T3SS, T4SS, and T6SS secretory systems. EffectiveDB also identified eukaryotic-like domains, potential T3SS, and potential T4SS secreted proteins in the MSCT1 predicted proteome. Additionally, blastp was used to align the proteins of the MSCT1 predicted proteome to the 502 proteins representing 53 effector families of *Xanthomonas* species found in the *Xanthomonas* effector database (Xanthomonas.org) [34]. Transcription activator-like effectors and Repeat Variable Diresidues were predicted with AnnoTALE [44]. TALgetter [45] was used to identify the DNA target domain on the *G. hirsutum* line TM − 1 promoterome [46].

## Genome properties

**Fig. 4:**
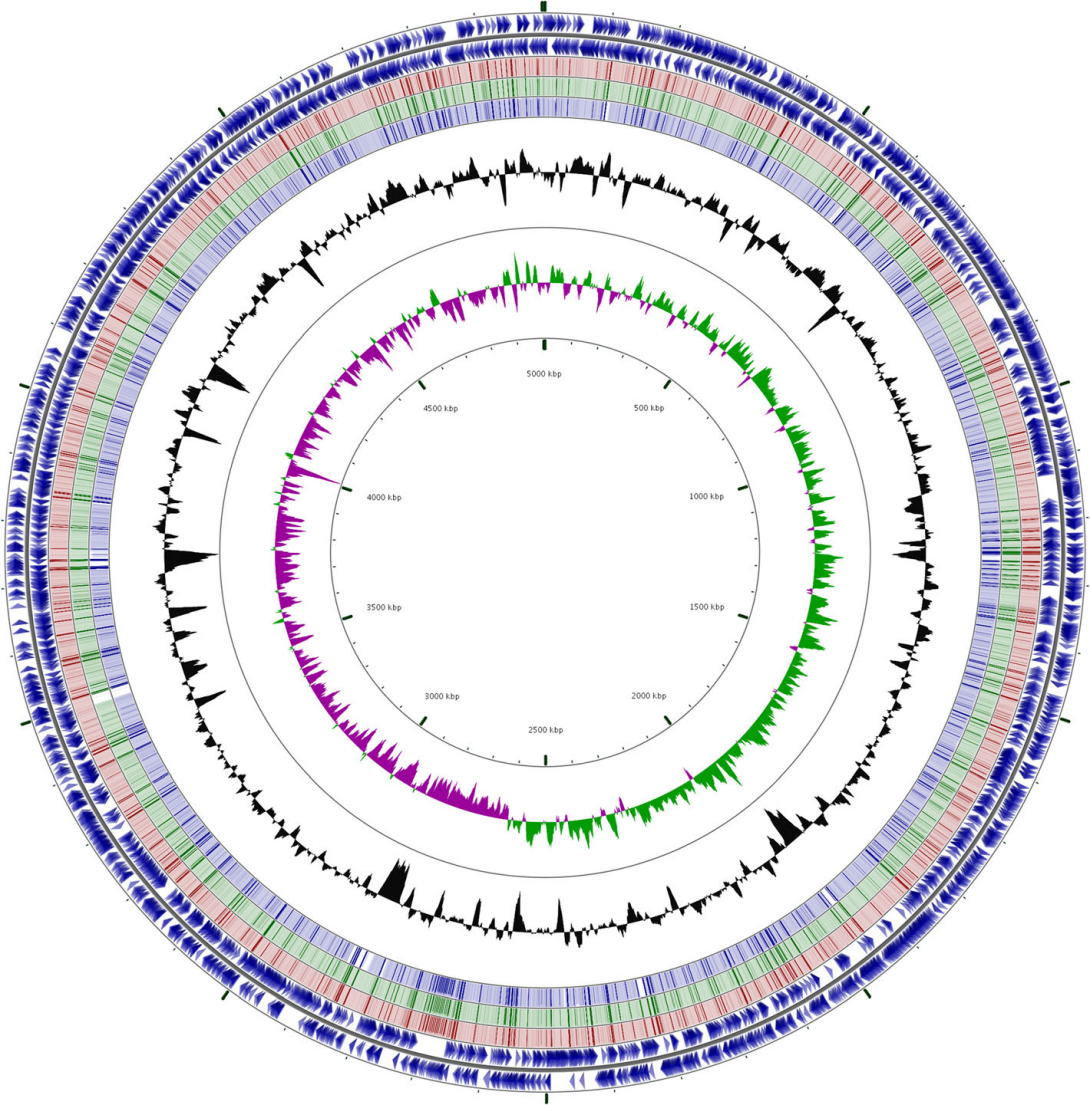
The genomic map of MSCT1 Chromosome 1. The outer and inner *dark blue* rings represents protein coding genes on the (+) and (−) strands, respectively. The light red, green and *blue* rings represent blastn alignments to MSCT1 against *X. citri* pv. *malvacearum* strains; R18 from Nicaragua (GCF_000309905.1), R18 from Burkina Faso (GCF_000454505.1), R20 from Burkina Faso (GCF_000454525.1), respectively. The *black* ring represents the gc content, while the inner green and purple ring represents the gc skew. The genomic map was created with cgview [88]

**Fig. 5:**
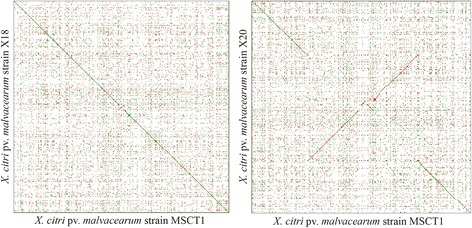
*Dot* plot of *X. citri* pv. *malvacearum* strain MSCT1 chromosome (NZ_CP017020.1) (X-Axis) compared to *X. citri* pv. *malvacearum* strain X18 (NZ_CM002136.1) (*left*, Y-Axis) and *X. citri* pv. *malvacearum* strain X20 (NZ_CM002029.1) (*right*, Y-Axis) Chromosomes. Dot plot produced with YASS web server using default settings [89]

The MSCT1 long read assembly had a sum length of 5,123,946 bp distributed along one large circular chromosome 5 Mb (Fig. 4) in length and 3 circular plasmids (60, 44, and 15 kb in length) (Table 3). Sequencing depth was 558.48 genome equivalents for the long read sequencing technology and 1820.26 genome equivalents for the Illumina PCR-free DNA library (Table 2). Dot plots determined the MSCT1 chromosome exhibited a high degree of sequence similarity to the circular chromosomes reported in previous *X. citri* pv. *malvacearum* assemblies (Fig. 5). A total of 4410 genes were predicted for MSCT1 including 4102 protein coding, 95 rRNA, and 213 pseudogenes (Table 4). The NCBI Prokaryotic Genome Annotation Pipeline added functional annotation to 2843 proteins.

**Table 3:** Summary of genome: one chromosome and 3 plasmids

Label	Size (Mb)	Topology	INSDC identifier	RefSeq ID
Chromosome	5.0	Circular	CP017020.1	NZ_CP017020.1
pMSCT15kb	15,263 (bp)	Circular	CP017021.1	NZ_CP017021.1
pMSCT44kb	43,946 (bp)	Circular	CP017022.1	NZ_CP017022.1
pMSCT60kb	60,533 (bp)	Circular	CP017023.1	NZ_CP017023.1

**Table 4:** Genome statistics

Attribute	Value	% of Total
Genome size (bp)	5,123,946	100.00
DNA coding (bp)	4,365,468	85.20
DNA G + C (bp)	3,313,791	64.67
DNA scaffolds	4	100.00
Total genes	4410	100.00
Protein coding genes	4102	93.02
RNA genes	95	2.15
Pseudo genes	213	4.83
Genes in internal clusters	-	-
Genes with function prediction	3375	76.53
Genes assigned to COGs	3228	73.20
Genes with Pfam domains	3302	74.88
Genes with signal peptides	553	12.54
Genes with transmembrane helices	911	20.66
CRISPR repeats	1	-

The predominate COG functional classifications were R (general function), E (amino acid transport and metabolism), M (cell wall/membrane biogenesis), and H (coenzyme transport and metabolism), representing 16.31, 11.68, 10.36, and 9.68% of the predicted proteome, respectively (Table 5). InterProScan identified 3302 proteins containing at least one Pfam domain. In total, 3375 proteins contained at least one functional annotation from either the Pfam or COG annotations (Table 4). The rRNA segments were comprised of two copies of each of the 23S, 5S, and 16S rRNA subunits. At least one tRNA for each of the 20 basic amino acids was identified in the 54 predicted tRNA loci. Transmembrane helices prediction identified 911 proteins with at least one predicted transmembrane helix. Signal peptides were identified on 553 proteins; of these, after in silico cleavage of the predicted signal peptide, 23 contained a predicted transmembrane helix leaving 530 proteins that can be secreted from the cell. A single CRISPR sequence with a sequence length of 298 bp was predicted in the genome assembly in the 27,394 to 27,692 bp region of the MSCT1 chromosome. As is common in species of *Xanthomonas* multiple copies of the transposase coding genes were identified dispersed throughout the genome [47]. In total 26 transpose genes were predicted in MSCT1, making it the fourth most abundant functional annotation in the proteome (Table 6).

**Table 5:** Number of genes associated with general COG functional categories

Code	Value	% age	Description
J	349	8.51	Translation, ribosomal structure and biogenesis
A	1	0.02	RNA processing and modification
K	348	8.48	Transcription
L	208	5.017	Replication, recombination and repair
B	3	0.07	Chromatin structure and dynamics
D	129	3.14	Cell cycle control, Cell division, chromosome partitioning
V	168	4.10	Defense mechanisms
T	344	8.39	Signal transduction mechanisms
M	425	10.36	Cell wall/membrane biogenesis
N	241	5.88	Cell motility
U	198	4.83	Intracellular trafficking and secretion
O	295	7.19	Posttranslational modification, protein turnover, chaperones
C	369	9.00	Energy production and conversion
G	366	8.92	Carbohydrate transport and metabolism
E	479	11.68	Amino acid transport and metabolism
F	129	3.14	Nucleotide transport and metabolism
H	397	9.68	Coenzyme transport and metabolism
I	266	6.48	Lipid transport and metabolism
P	358	8.72	Inorganic ion transport and metabolism
Q	243	5.92	Secondary metabolites biosynthesis, transport and catabolism
R	669	16.31	General function prediction only
S	263	6.41	Function unknown
-	874	21.31	Not in COGs

The total is based on the total number of protein coding genes in the genome

**Table 6:** Ten most represented functional annotations

Annotation	Count
Membrane protein	64
TonB-dependent receptor	42
MFS transporter	33
Transposase	26
Transcriptional regulator	25
ABC transporter ATP-binding protein	23
Oxidoreductase	19
LysR family transcriptional regulator	19
GGDEF domain-containing protein	16
Two-component sensor histidine kinase	15

## Insights from the genome sequence

Functional T3SS, T4SS, and T6SS secretory systems were predicted in MSCT1. Comparison of the MSCT1 predicted proteins with previously described *Xanthomonas* effectors resulted in the identification of 7 families of effectors common among species of *Xanthomonas* (Table 7). These classes include AvrBs2, XopAG, XopK, XopP, XopR, XopT, and XopZ1. Effectors AvrBs2, XopK, XopP, XopR, and XopZ1, have been shown to suppress the host disease resistance response and immunity in other plant-*Xanthomonas* interactions [48–54]. XopAG effector family members have been shown to be responsible for eliciting the hyper-sensitive response in grapefruit [55]. The predicted protein sequence WP_033481547.1, predicted from the MSCT1 genome, exhibited homology to AvrBs2 effector proteins from several species of *Xanthomonas* and contained a predicted glycerophosphoryl diester phosphodiesterase family (PF03009) domain characteristic of the AvrBs2 effector family [10]. AvrBs2 produced by *Xanthomonas campestris* pv. *vesicatoria* is recognized by a NBS-LRR in peppers containing the Bs2 resistance gene; however, field strains of *X. campestris* pv. *vesicatoria* have been identified that evade the recognition [56, 57].

**Table 7:** Xanthomad Non-TAL Effector families found in MSCT1

Family	Refseq_ID	BlastP HIT	Notes	REF
XopAG	WP_033479491.1	CAP49915.1	HR response in Grapefruit	[55]
XopK	WP_005915119.1	CAP50604.1	Unclear role in virulence	[52, 83, 84]
XopP	WP_069288200.1	CAJ22867.1	Suppresses immune response in rice	[49]
XopR	WP_005923840.1	BAE70889.1	Suppression of MAMP-triggered immune responses	[48, 53, 54]
XopT	WP_069288215.1	BAE68965.1	-	[83]
XopZ1	WP_005914471.1	BAE69157.1	Contributes to virulence in rice	[51, 52]
AvrBs2	WP_033481547.1	CAJ21683.1	Suppresses rice immunity	[50]

EffectiveDB predicted 408 T3SS and 44 T4SS secreted proteins. MSCT1 predicted secreted proteins that have previously been associated with diseases in *G. hirsutum* and other plant systems include; endoglucanase [58], polygalacturonase [59], glutathione S-transferase [60], pectate lyase [61], glutathione peroxidase [62], as well as catabolic enzymes such as peptidases and lipases. These protein likely aid the mediation of the host disease response as well as the breaking down of host tissues. The PIP-box sequence was identified 78 bp up stream of the start codon for the *HrpB1* gene, that indicates gene regulation via PIP targeted transcription factors are present in the MSCT1 genome. EffectiveDB also identified 22 eukaryotic-like domains among 36 MSCT1 proteins. The most represented eukaryotic-like domains were the of M13 peptidase family (PF01431 and PF05649); however, M13 peptidases are commonly identified among bacteria [63].

## Extended insights

AnnoTAL identified 8 potential CDS regions in the MSCT1 genome that could potentially code for TAL effectors (Table 8). AnnoTAL did not predict any TAL sequences in the other four draft *X. citri* pv. *malvacearum* genomes reported previously (GCF_000454505.1 (strain: X18), GCF_000454525.1 (strain: X20), GCF_000309925.1 (strain: GSPB22388) and GCF_000309915.1 (strain: GSPB1386)). Interestingly, 7 of the 8 TAL effectors in *X. citri* pv. *malvacearum* MSCT1 are located on plasmids. This arrangement is in contrast to other xanthomonads such as *Xanthomonas oryzae* pv. *oryzae* and *Xanthomonas oryzae* pv. *oryzicola* where the vast majority of TAL effectors are located on the large chromosome. The presence of the *X. citri* pv. *malvacearum* TAL effectors in *X. citri* pv. *malvacearum* plasmids can be traced back to the initial report by De Feyter et al. 1991 [64], that described 6 avirulence genes on a 90 kb *X. citri* pv. *malvacearum* plasmid [20–22]. However, the *X. citri* species and *X. oryzae* species such as *X. oryzae* pv. *oryzae* and *X. oryzae* pv. *oryzicola* exhibit evolutionarily divergence and fall into different clades among the other sequenced xanthomonads in phylogenic analysis [65]. Although, the overall total number of TAL effectors found in MSCT1 (*n* = 8) is less than what has been previously reported for some *X. oryzae* pv. *oryzae* and *X. oryzae* pv. *oryzicola* strains it is similar to strains of *X. translucens* [43, 47, 66].

**Table 8:** MSCT1 Potential TAL Effectors

TAL	Molecule	Refseq_ID	Start^a^	Stop^a^	Strand^a^
MSCT1-TAL1	pMSCT44kb	WP_069288206.1	36,431	40,333	1
MSCT1-TAL2	pMSCT60kb	WP_069288209.1	16,043	19,127	1
MSCT1-TAL3	Chromosome	WP_069288181.1^b^	2,568,181	2,571,268	-1
MSCT1-TAL4	pMSCT44kb	WP_069288204.1	15,111	19,626	-1
MSCT1-TAL5	pMSCT60kb	WP_069288212.1	41,404	44,689	-1
MSCT1-TAL6	pMSCT60kb	WP_069288211.1	44,689	34,259	-1
MSCT1-TAL7	pMSCT60kb	WP_069288210.1	21,870	21,870	-1
MSCT1-TAL8	pMSCT60kb	WP_069288208.1	3549	8064	-1

^a^Start, Stop, and Strand annotations by AnnoTAL

^b^NCBI Annotation differs from AnnoTAL prediction, the MSCT1-TAL3 NCBI Start Codon begins at 2,570,908

The variable dinucleotide repeats were identified in the 8 MSCT1 TAL sequences for recognition of the TAL DNA target domain with the previously reported TAL code (Table 9). Due to the inherit degeneracy nature of TAL DTD prediction [12, 45, 67–70], potential TAL DTDs reported in this study are limited to the top 2 DTD site predictions for each TAL with the additional constraint of being within 150 bp of the gene start codon. Interestingly, MSCT1 TALs (MSCT1-TAL2 and MSCT1-TAL8) with a DTD prediction had predictions on corresponding sections of the A and D sub-genomes of the *G. hirsutum* TM − 1 assembly [46]. However, these in silico predictions still need to be confirmed with RNA expression data from studies of *G. hirsutum* undergoing infection by MSCT1. Of note, no MSCT1 TAL DTD was predicted to target any promoter region on *G. hirsutum* chromosome 14 or 20 that contain the *B*_2_, *B*_3_ and *B*_12_ genes that are a major source of resistance to *X. citri* pv. *malvacearum* [71–73].

**Table 9:** Repeat Variable Diresidues of MSCT1 TAL effectors

TAL	Repeat Variable Diresidues
MSCT1-TAL1	HD-NI-NG-NI-NI-NS-NG-NG-NI-NG-NS-HD-NS-HD-NS-NG-NS-NG-HD-NG-NG-NG
MSCT1-TAL2	NI-NI-NI-NN-NI-NS-HD-NG-HD-NS-NG-HD-HD-NG
MSCT1-TAL3	NI-NG-NI-HD-NG-NG-NG-NG-HD-NS-HD-HD-NG-NG
MSCT1-TAL4	NI-NG-NI-NG-NS-NS-NS-NG-HD-NS-HD-HD-HD-HD-HD-NG-NI-NG-NS-NG-NS-HD-HD-HD-HD-NG-NG-NG
MSCT1-TAL5	NI-NI-NI-NN-NI-NS-HD-NG-NN-NS-NN-NN-HD-NG-N*-NN
MSCT1-TAL6	NI-NG-NI-NI-NI-NG-NG-NS-NG-NS-NS-NG-NS-NG-HD-NS-HD-HD-NG-NS-NG-NG-NG-NG-NG-NG
MSCT1-TAL7	HD-NI-NG-NI-NI-NI-HD-HD-HD-NS-NS-HD-HD-NS-NS-NG-NS-NG-NG
MSCT1-TAL8	NI-NG-NI-NI-NI-NG-HD-HD-NS-NI-HD-NI-HD-HD-NI-NS-NG-HD-NS-NS-NS-NG-NS-NG-NG-NG-NG-NG

Of the predicted TALs only two, MSCT1-TAL2 and MSCT1-TAL8, had target sequences that fall within 100 bp of the start codon. MSCT1-TAL2 was predicted to target 21 bp from the start codon of the two paralogous proteins (Gh_A04G1143, Gh_D04G1757) found on chromosome 4 of each of the respective sub-genomes of tetraploid cotton. The proteins that MSCT1-TAL2 potential targets contain the ProSiteProfiles NAC domain profile (PS51005). The NAC domain has been reported to participate in both biotic and abiotic stress related responses [74]. MSCT1-TAL8 targeted 19 and 20 base pairs upstream of the paralogous proteins (Gh_A01G1702, Gh_D01G1952) in the A and D sub-genomes of *G. hirsutum*, respectively.

## Conclusions

The MSCT1 genome reported in this study is the first *X. citri* pv. *malvacearum* genome to be completed with long read DNA sequencing technology. The long read sequencing and assembly strategy allowed for the identification of eight TAL effectors in *X. citri* pv. *malvacearum* and makes the MSCT1 genome assembly the only *X. citri* pv. *malvacearum* genome with assembled TAL effectors. In addition to the TAL effector identification, many T3SS effectors were identified in MSCT1 genome. The genome assembly, as outlined in this paper, provides a basis for future epidemiological and pathogenesis studies of the *X. citri* pv. *malvacearum-G. hirsutum* pathogen host complex.

## Abbreviations

(DTD): DNA target domain
(HVS): Highly virulent strain
(MLST): Multilocus sequence typing
(PIP): Plant inducible promoter
(TAL): Transcription activator-like

## References

[1] Hillocks R, Hillocks R (1992). Bacterial blight. Cotton diseases.

[2] Morgham AT, Richardson PE, Essenberg M, Cover EC (1988). Effects of continuous dark upon ultrastructure, bacterial populations and accumulation of phytoalexins during interactions between *Xanthomonas campestris* pv. *malvacearum* and bacterial blight susceptible and resistant cotton. Physiol Mol Plant Pathol.

[3] Al Mousawi A, Richardson P, Essenberg M, Johnson W. Ultrastructural studies of a compatible interaction between *Xanthomonas campestris* pv. *malvacearum* and cotton [*Gossypium hirsutum*, bacterial blight]. Phytopathology. 1982;72:1222-30.

[4] Rudolph K, Swings JG, Civerolo EL (1993). Infection of the plant by *Xanthomonas*. Xanthomonas.

[5] Knight R, Clouston T (1939). The genetics of blackarm resistance. J Genet.

[6] Bourland F, Myers GO (2015). Conventional cotton breeding. Cotton.

[7] Bourland FM, Jones DC (2013). Registration of ‘UA103’ cotton cultivar. J Plant Registrations.

[8] Bourland FM, Jones DC (2015). Registration of Arkot 0305, Arkot 0306, Arkot 0309, and Arkot 0316 germplasm lines of cotton. J Plant Registrations.

[9] Delannoy E, Lyon BR, Marmey P, Jalloul A, Daniel JF, Montillet JL, Essenberg M, Nicole M (2005). Resistance of cotton towards *Xanthomonas campestris* pv. *malvacearum*. Annu Rev Phytopathol.

[10] Buttner D, Bonas U (2010). Regulation and secretion of *Xanthomonas* virulence factors. FEMS Microbiol Rev.

[11] Zhang J, Yin Z, White F (2015). TAL effectors and the executor R genes. Front Plant Sci.

[12] Boch J, Bonas U, Lahaye T (2014). TAL effectors – pathogen strategies and plant resistance engineering. New Phytol.

[13] Mak AN-S, Bradley P, Bogdanove AJ, Stoddard BL (2013). TAL effectors: function, structure, engineering and applications. Curr Opin Struct Biol.

[14] Cunnac S, Bolot S, Forero Serna N, Ortiz E, Szurek B, Noel LD, Arlat M, Jacques MA, Gagnevin L, Carrere S, et al. High-quality draft genome sequences of two *Xanthomonas citri* pv. *malvacearum* strains. Genome Announc. 2013;1(4):1–2. 10.1128/genomeA.00674-13 PMC3757453 23990578

[15] Zhai J, Xia Z, Liu W, Jiang X, Huang X (2013). Genomic sequencing globally identifies functional genes and potential virulence-related effectors of *Xanthomonas axonopodis* pv. *malvacearum*. Eur J Plant Pathol.

[16] National Cotton Council Disease Database. National Cotton Council of America. 2013. http://www.cotton.org/tech/pest/upload/Disease-Database-2013-2.xls. Accessed 25 Jan 2017.

[17] Ah-You N, Gagnevin L, Grimont PA, Brisse S, Nesme X, Chiroleu F, Bui Thi Ngoc L, Jouen E, Lefeuvre P, Verniere C (2009). Polyphasic characterization of xanthomonads pathogenic to members of the Anacardiaceae and their relatedness to species of *Xanthomonas*. Int J Syst Evol Microbiol.

[18] Gross DC, DeVay JE (1977). Production and purification of syringomycin, a phytotoxin produced by *Pseudomonas syringae*. Physiol Plant Pathol.

[19] Xu J, Deng P, Showmaker KC, Wang H, Baird SM, Lu S-E (2014). The pqqC gene is essential for antifungal activity of *Pseudomonas kilonensis* JX22 against *Fusarium oxysporum* f. Sp. *lycopersici*. FEMS Microbiol Lett.

[20] Yang Y, De Feyter R, Gabriel DW (1994). Host-specific symptoms and increased release of *Xanthomonas citri* and *X. campestris* pv. *malvacearum* from leaves are determined by the 102-bp tandem repeats of pthA and avrb6, respectively. Mol Plant Microbe Interact.

[21] Yang Y, Gabriel DW (1995). *Xanthomonas* avirulence/pathogenicity gene family encodes functional plant nuclear targeting signals. Mol Plant Microbe Interact.

[22] Boch J, Bonas U (2010). *Xanthomonas* AvrBs3 family-type III effectors: discovery and function. Annu Rev Phytopathol.

[23] Chen WP, Kuo TT (1993). A simple and rapid method for the preparation of gram-negative bacterial genomic DNA. Nucleic Acids Res.

[24] Koren S, Walenz BP, Berlin K, Miller JR, Bergman NH, Phillippy AM. Canu: scalable and accurate long-read assembly via adaptive k-mer weighting and repeat separation. Genome Res. 2017;27:722–36. 10.1101/gr.215087.116 PMC5411767 28298431

[25] Rice P, Longden I, Bleasby A (2000). EMBOSS: the European molecular biology open software suite. Trends Genet.

[26] Altschul SF, Gish W, Miller W, Myers EW, Lipman DJ (1990). Basic local alignment search tool. J Mol Biol.

[27] Chaisson MJ, Tesler G (2012). Mapping single molecule sequencing reads using basic local alignment with successive refinement (BLASR): application and theory. BMC Bioinformatics.

[28] Robinson JT, Thorvaldsdottir H, Winckler W, Guttman M, Lander ES, Getz G, Mesirov JP (2011). Integrative genomics viewer. Nat Biotech.

[29] Thorvaldsdóttir H, Robinson JT, Mesirov JP (2013). Integrative genomics viewer (IGV): high-performance genomics data visualization and exploration. Brief Bioinform.

[30] Bolger AM, Lohse M, Usadel B (2014). Trimmomatic: a flexible trimmer for Illumina sequence data. Bioinformatics.

[31] Walker BJ, Abeel T, Shea T, Priest M, Abouelliel A, Sakthikumar S, Cuomo CA, Zeng Q, Wortman J, Young SK (2014). Pilon: an integrated tool for comprehensive microbial variant detection and genome assembly improvement. PLoS One.

[32] Field D, Garrity G, Gray T, Morrison N, Selengut J, Sterk P, Tatusova T, Thomson N, Allen MJ, Angiuoli SV (2008). The minimum information about a genome sequence (MIGS) specification. Nat Biotechnol.

[33] Tatusova T, DiCuccio M, Badretdin A, Chetvernin V, Nawrocki EP, Zaslavsky L, Lomsadze A, Pruitt KD, Borodovsky M, Ostell J (2016). NCBI prokaryotic genome annotation pipeline. Nucleic Acids Res.

[34] Camacho C, Coulouris G, Avagyan V, Ma N, Papadopoulos J, Bealer K, Madden TL (2009). BLAST+: architecture and applications. BMC Bioinformatics.

[35] Galperin MY, Makarova KS, Wolf YI, Koonin EV (2015). Expanded microbial genome coverage and improved protein family annotation in the COG database. Nucleic Acids Res.

[36] Marchler-Bauer A, Derbyshire MK, Gonzales NR, Lu S, Chitsaz F, Geer LY, Geer RC, He J, Gwadz M, Hurwitz DI (2015). CDD: NCBI’s conserved domain database. Nucleic Acids Res.

[37] Quevillon E, Silventoinen V, Pillai S, Harte N, Mulder N, Apweiler R, Lopez R (2005). InterProScan: protein domains identifier. Nucleic Acids Res.

[38] Petersen TN, Brunak S, von Heijne G, Nielsen H (2011). SignalP 4.0: discriminating signal peptides from transmembrane regions. Nat Methods.

[39] Krogh A, Larsson B, von Heijne G, Sonnhammer EL (2001). Predicting transmembrane protein topology with a hidden Markov model: application to complete genomes. J Mol Biol.

[40] Grissa I, Vergnaud G, Pourcel C (2007). CRISPRFinder: a web tool to identify clustered regularly interspaced short palindromic repeats. Nucleic Acids Res.

[41] Fenselau S, Bonas U (1995). Sequence and expression analysis of the hrpB pathogenicity operon of *Xanthomonas campestris* pv. *vesicatoria* which encodes eight proteins with similarity to components of the Hrp, Ysc, spa, and Fli secretion systems. Mol Plant Microbe Interact.

[42] Lee B-M, Park Y-J, Park D-S, Kang H-W, Kim J-G, Song E-S, Park I-C, Yoon U-H, Hahn J-H, Koo B-S (2005). The genome sequence of *Xanthomonas oryzae* pathovar *oryzae* KACC10331, the bacterial blight pathogen of rice. Nucleic Acids Res.

[43] Peng Z, Hu Y, Xie J, Potnis N, Akhunova A, Jones J, Liu Z, White FF, Liu S (2016). Long read and single molecule DNA sequencing simplifies genome assembly and TAL effector gene analysis of *Xanthomonas translucens*. BMC Genomics.

[44] Grau J, Reschke M, Erkes A, Streubel J, Morgan RD, Wilson GG, Koebnik R, Boch J (2016). AnnoTALE: bioinformatics tools for identification, annotation, and nomenclature of TALEs from *Xanthomonas* genomic sequences. Sci Rep.

[45] Grau J, Wolf A, Reschke M, Bonas U, Posch S, Boch J (2013). Computational predictions provide insights into the biology of TAL effector target sites. PLoS Comput Biol.

[46] Zhang T, Hu Y, Jiang W, Fang L, Guan X, Chen J, Zhang J, Saski CA, Scheffler BE, Stelly DM (2015). Sequencing of allotetraploid cotton (*Gossypium hirsutum* L. acc. TM−1) provides a resource for fiber improvement. Nat Biotech.

[47] Booher NJ, Carpenter SCD, Sebra RP, Wang L, Salzberg SL, Leach JE, Bogdanove AJ. Single molecule real-time sequencing of *Xanthomonas oryzae* genomes reveals a dynamic structure and complex TAL (transcription activator-like) effector gene relationships. Microbial Genomics. 2015;1(4):1–22. 10.1099/mgen.0.000032 PMC4853030 27148456

[48] Akimoto-Tomiyama C, Furutani A, Tsuge S, Washington EJ, Nishizawa Y, Minami E, Ochiai H (2011). XopR, a type III effector secreted by *Xanthomonas oryzae* pv. *oryzae*, suppresses microbe-associated molecular pattern-triggered immunity in *Arabidopsis thaliana*. Mol Plant-Microbe Interact.

[49] Ishikawa K, Yamaguchi K, Sakamoto K, Yoshimura S, Inoue K, Tsuge S, Kojima C, Kawasaki T. Bacterial effector modulation of host E3 ligase activity suppresses PAMP-triggered immunity in rice. Nat Commun. 2014;5:1–11. 10.1038/ncomms6430 25388636

[50] Li S, Wang Y, Wang S, Fang A, Wang J, Liu L, Zhang K, Mao Y, Sun W (2015). The type III effector AvrBs2 in *Xanthomonas oryzae* pv. Oryzicola suppresses rice immunity and promotes disease development. Mol Plant-Microbe Interact.

[51] Sinha D, Gupta MK, Patel HK, Ranjan A, Sonti RV (2013). Cell wall degrading enzyme induced rice innate immune responses aresuppressed by the type 3 secretion system effectors XopN, XopQ, XopX and XopZ of *Xanthomonas oryzae* pv. *oryzae*. PLoS One.

[52] Song C, Yang B (2010). Mutagenesis of 18 type III effectors reveals virulence function of XopZPXO99 in *Xanthomonas oryzae* pv. *oryzae*. Mol Plant-Microbe Interact.

[53] Wang S, Sun J, Fan F, Tan Z, Zou Y, Lu D (2016). A *Xanthomonas oryzae* pv. *oryzae* effector, XopR, associates with receptor-like cytoplasmic kinases and suppresses PAMP-triggered stomatal closure. Sci China Life Sci.

[54] Zhao S, Mo W-L, Wu F, Tang W, Tang J-L, Szurek B, Verdier V, Koebnik R, Feng J-X (2013). Identification of non-TAL effectors in *Xanthomonas oryzae* pv. *oryzae* Chinese strain 13751 and analysis of their role in the bacterial virulence. World J Microbiol Biotechnol.

[55] Escalon A, Javegny S, Vernière C, Noël LD, Vital K, Poussier S, Hajri A, Boureau T, Pruvost O, Arlat M (2013). Variations in type III effector repertoires, pathological phenotypes and host range of *Xanthomonas citri* pv. *citri* pathotypes. Mol Plant Pathol.

[56] Gassmann W, Dahlbeck D, Chesnokova O, Minsavage GV, Jones JB, Staskawicz BJ (2000). Molecular evolution of virulence in natural field strains of *Xanthomonas campestris* pv. *vesicatoria*. J Bacteriol.

[57] Tai TH, Dahlbeck D, Clark ET, Gajiwala P, Pasion R, Whalen MC, Stall RE, Staskawicz BJ (1999). Expression of the Bs2 pepper gene confers resistance to bacterial spot disease in tomato. Proc Natl Acad Sci U S A.

[58] Wubben MJ, Ganji S, Callahan FE (2010). Identification and molecular characterization of a β−1,4-endoglucanase gene (Rr-eng-1) from *Rotylenchulus reniformis*. J Nematol.

[59] Showmaker KC, Bednárová A, Gresham C, Hsu C-Y, Peterson DG, Krishnan N (2016). Insight into the salivary gland transcriptome of *Lygus lineolaris* (Palisot de Beauvois). PLoS One.

[60] Espada M, Jones JT, Mota M (2016). Characterization of glutathione S-transferases from the pine wood nematode. Nematology.

[61] Danchin EGJ, Rosso M-N, Vieira P, de Almeida-Engler J, Coutinho PM, Henrissat B, Abad P (2010). Multiple lateral gene transfers and duplications have promoted plant parasitism ability in nematodes. Proc Natl Acad Sci.

[62] Jones JT, Reavy B, Smant G, Prior AE (2004). Glutathione peroxidases of the potato cyst nematode *Globodera Rostochiensis*. Gene.

[63] Bianchetti L, Oudet C, Poch O (2002). M13 endopeptidases: new conserved motifs correlated with structure, and simultaneous phylogenetic occurrence of PHEX and the bony fish. Proteins.

[64] De Feyter R, Gabriel DW (1991). At least six avirulence genes are clustered on a 90-kilobase plasmid in *Xanthomonas campestris* pv. *malvacearum*. Mol Plant-Microbe Interact.

[65] Rodriguez-R LM, Grajales A, Arrieta-Ortiz ML, Salazar C, Restrepo S, Bernal A (2012). Genomes-based phylogeny of the genus *Xanthomonas*. BMC Microbiol.

[66] Hersemann L, Wibberg D, Widmer F, Vorhölter F-J, Kölliker R (2016). Draft genome sequences of three *Xanthomonas translucens* pathovar reference strains (pv. *arrhenatheri*, pv. *poae* and pv. *phlei*) with different specificities for forage grasses. Stand Genomic Sci.

[67] Cernadas RA, Doyle EL, Niño-Liu DO, Wilkins KE, Bancroft T, Wang L, Schmidt CL, Caldo R, Yang B, White FF (2014). Code-assisted discovery of TAL effector targets in bacterial leaf streak of rice reveals contrast with bacterial blight and a novel susceptibility gene. PLoS Pathog.

[68] Moscou MJ, Bogdanove AJ (2009). A simple cipher governs DNA recognition by TAL effectors. Science (New York, NY).

[69] Boch J, Scholze H, Schornack S, Landgraf A, Hahn S, Kay S, Lahaye T, Nickstadt A, Bonas U. Breaking the code of DNA binding specificity of TAL-type III effectors. Science (New York, NY). 326(5959):2009, 1509–1512. 10.1126/science.1178811 19933107

[70] Booher NJ, Bogdanove AJ (2014). Tools for TAL effector design and target prediction. Methods.

[71] Silva RA, Barroso PAV, Hoffmann LV, Giband M, Coutinho WM (2014). A SSR marker linked to the B 12 gene that confers resistance to race 18 of *Xanthomonas axonopodis* pv. *malvacearum* in cotton is also associated with other bacterial blight resistance gene complexes. Australas Plant Pathol.

[72] Wallace TP, El-Zik KM (1989). Inheritance of resistance in three cotton cultivars to the HV1 isolate of bacterial blight. Crop Sci.

[73] Wright RJ, Thaxton PM, El-Zik KM, Paterson AH (1998). D-subgenome bias of *Xcm* resistance genes in tetraploid *Gossypium* (cotton) suggests that polyploid formation has created novel avenues for evolution. Genetics.

[74] Nuruzzaman M, Sharoni AM, Kikuchi S (2013). Roles of NAC transcription factors in the regulation of biotic and abiotic stress responses in plants. Front Microbiol.

[75] Field D, Amaral-Zettler L, Cochrane G, Cole JR, Dawyndt P, Garrity GM, Gilbert J, Glöckner FO, Hirschman L, Karsch-Mizrachi I (2011). The genomic standards consortium. PLoS Biol.

[76] Woese CR, Kandler O, Wheelis ML (1990). Towards a natural system of organisms: proposal for the domains Archaea, bacteria, and Eucarya. Proc Natl Acad Sci U S A.

[77] Garrity GM, Bell JA, Lilburn T, Garrity GM, Brenner DJ, Krieg NR, Staley JT (2005). Phylum XIV. Proteobacteria phyl. Nov. Bergey’s manual of systematic bacteriology volume 2, part B.

[78] Garrity GM, Bell JA, Class LT, Garrity GM, Brenner DJ, Krieg NR, Staley JT (2005). Gammaproteobacteria class. Nov. Bergey’s manual of systematic bacteriology volume 2, part B.

[79] Euzéby J. Validation of publication of new names and new combinations previously effectively published outside the IJSEM. List no. 106. Int J Syst Evol Microbiol. 2005;55:2235–8. 10.1099/ijs.0.63767-0 15879221

[80] Dowson D (1939). On the systematic position and generic names of the gram negative bacterial plant pathogens. Zentralblatt fur Bakteriologie, Parasitenkunde und Infektionskrankheiten, 2.

[81] Hayward A, Waterson C (1964). Xanthomonas malvacearum.

[82] Ashburner M, Ball CA, Blake JA, Botstein D, Butler H, Cherry JM, Davis AP, Dolinski K, Dwight SS, Eppig JT (2000). Gene ontology: tool for the unification of biology. The gene ontology consortium. Nat Genet.

[83] Furutani A, Takaoka M, Sanada H, Noguchi Y, Oku T, Tsuno K, Ochiai H, Tsuge S (2008). Identification of novel type III secretion effectors in *Xanthomonas oryzae* pv. *oryzae*. Mol Plant-Microbe Interact.

[84] Mutka AM, Fentress SJ, Sher JW, Berry JC, Pretz C, Nusinow DA, Bart R (2016). Quantitative, image-based phenotyping methods provide insight into spatial and temporal dimensions of plant disease. Plant Physiol.

[85] Katoh K, Standley DM (2013). MAFFT multiple sequence alignment software version 7: improvements in performance and usability. Mol Biol Evol.

[86] Tamura K, Nei M (1993). Estimation of the number of nucleotide substitutions in the control region of mitochondrial DNA in humans and chimpanzees. Mol Biol Evol.

[87] Tamura K, Stecher G, Peterson D, Filipski A, Kumar S (2013). MEGA6: molecular evolutionary genetics analysis version 6.0. Mol Biol Evol.

[88] Grant JR, Stothard P (2008). The CGView server: a comparative genomics tool for circular genomes. Nucleic Acids Res.

[89] Noé L, Kucherov G (2005). YASS: enhancing the sensitivity of DNA similarity search. Nucleic Acids Res.

